# Back-illuminated photoelectrochemical flow cell for efficient CO_2_ reduction

**DOI:** 10.1038/s41467-022-34926-x

**Published:** 2022-11-19

**Authors:** Bin Liu, Tuo Wang, Shujie Wang, Gong Zhang, Dazhong Zhong, Tenghui Yuan, Hao Dong, Bo Wu, Jinlong Gong

**Affiliations:** 1 School of Chemical Engineering and Technology; Key Laboratory for Green Chemical Technology of Ministry of Education, Tianjin University, Tianjin, 300072 China; 2 Collaborative Innovation Center of Chemical Science and Engineering (Tianjin), Tianjin, 300072 China; 3 Haihe Laboratory of Sustainable Chemical Transformations, Tianjin, 300192 China; 4 Joint School of National University of Singapore and Tianjin University, International Campus of Tianjin University, Binhai New City, Fuzhou, 350207 China

**Keywords:** Electrocatalysis, Materials for energy and catalysis, Chemical engineering

## Abstract

Photoelectrochemical CO_2_ reduction reaction flow cells are promising devices to meet the requirements to produce solar fuels at the industrial scale. Photoelectrodes with wide bandgaps do not allow for efficient CO_2_ reduction at high current densities, while the integration of opaque photoelectrodes with narrow bandgaps in flow cell configurations still remains a challenge. This paper describes the design and fabrication of a back-illuminated Si photoanode promoted PEC flow cell for CO_2_ reduction reaction. The illumination area and catalytic sites of the Si photoelectrode are decoupled, owing to the effective passivation of defect states that allows for the long minority carrier diffusion length, that surpasses the thickness of the Si substrate. Hence, a solar-to-fuel conversion efficiency of CO of 2.42% and a Faradaic efficiency of 90% using Ag catalysts are achieved. For CO_2_ to C_2+_ products, the Faradaic efficiency of 53% and solar-to-fuel of 0.29% are achieved using Cu catalyst in flow cell.

## Abstract (web summary)

Conversion of CO_2_ into value-added products with renewable energy provides a promising route to realize carbon neutrality. Here, authors design and construct a Si photoelectrochemical flow cells for CO_2_ reduction reaction to achieve a high solar-to-fuel conversion efficiency.

## Introduction

Photoelectrochemical (PEC) reduction of CO_2_ use sunlight to directly drive the CO_2_RR for solar fuel production^1–3^. PEC CO_2_ reduction systems, that utilize photocathode^4,5^ and photoanode^6,7^ driven systems, have been widely investigated. Photocathodes can employ CO_2_RRs catalysts to carry out the CO_2_RR in H-type cells, where the cell voltage could be supplemented by photovoltage, thus lowering the external energy input by photovoltage generated by photoelectrode^8–10^. However, many photocathodes suffer from low activity or poor stability for PEC CO_2_RR, because the catalyst/semiconductor interface tends to produce hydrogen and suffers from photo-corrosion in aqueous electrolytes^2,5,11–14^. Furthermore, the deployment of photocathodes in flow cell configurations for PEC CO_2_ reduction remains a challenge because most high-performance semiconductor materials exist as dense layers, for which a compact substrate is always needed. Thus, it is a great challenge to simultaneously use semiconductors as a gas diffusion layer and sunlight absorber. On the other hand, photoanodes such as TiO_2_^15,16^, WO_3_^7^, BiVO_4_^7,17^, GaAs^18^, and Si^19^ have been integrated to form PEC CO_2_ reduction cells, and exhibit a reduced cell voltage for CO_2_ reduction. However, there is still room to increase the current density, because many photoanode-assisted PEC CO_2_RR were all carried out in H-type cell^20,21^. The photoanode promoted flow cell for PEC CO_2_ reduction is a promising way to improve the energy utilization efficiency.

One of the prerequisites for flow cell photoelectrode operation is for the photoelectrode to generate a significant photocurrent, allowing the reduction reaction to proceed at a high rate. Irtem et al. elegantly pioneered a TiO_2_ photoanode-assisted flow cell for the reduction of CO_2_ to HCOO^−^, which demonstrated the feasibility of employing photoelectrodes within flow cells^22^. In this configuration, however, only photoanodes with wide bandgaps such as TiO_2_^23,24^, Fe_2_O_3_^25^, WO_3_^26^, BiVO_4_^27^, etc. could only be used since light could be introduced from the back side of the photoanode through the FTO substrate, leaving the front side facing the cathode for reaction. The relatively small photocurrent density of wide bandgap photoanodes limits the reaction rate and changes the CO_2_ reduction product distribution. Therefore, photoelectrodes capable of generating high photocurrents are highly desired for PEC CO_2_RR flow cells.

Silicon (Si), a narrow bandgap photoabsorber, is a promising photoanode candidate to drive the PEC CO_2_RR in flow cells due to its high saturation current density (larger than 40 mA/cm^2^)^4,28–31^. Moreover, single-junction Si solar cells are cheaper and have simpler fabrication processes than multi-junction solar cells designed for outer space applications^18,32,33^. However, most conventional Si photoanodes operate in the front-illumination mode (where illumination occurs on the same side as the catalysts and junction) because of its opaque nature, which makes it difficult to introduce light from its back side, and thus cannot be adapted to flow cell configurations^34–36^. Si with back-illuminated configurations is an elegant solution to this conundrum, as they decouple the location of light absorption and surface reaction sites^37–39^. Therefore, the implementation of back-illuminated Si photoelectrodes into PEC CO_2_RR flow cells has the potential to improve their energy utilization efficiency.

This paper describes the design and realization of a PEC CO_2_RR system that integrates a Si-based narrow bandgap photoelectrode to convert solar energy into chemicals at high photocurrent densities. The light absorption and catalytic sites could be separated onto different sides of the opaque Si photoelectrode, eliminating the tradeoff between optimized light absorption and efficient CO_2_ reduction in flow cells. The Si photoelectrode-assisted flow cell affords a high Faradaic efficiency of 90% and 53% for CO or C_2+_ products respectively, achieving the solar-to-fuel (STF) conversion efficiency of 2.42% for CO products. This work provides a promising design strategy for Si-based solar fuel production.

## Results

### Configuration of PEC flow cell for CO_2_RR with a double-sided Si photoanode

**Fig. 1:**
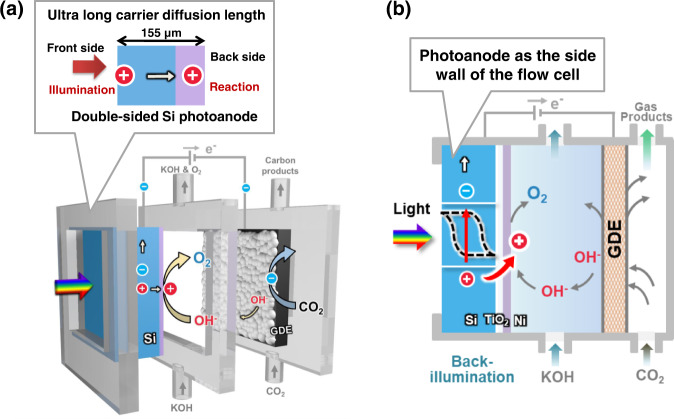
Si photoelectrode promoted flow cell. **a** The schematic of the Si photoanode coupled flow cell with the gas diffusion electrode for PEC CO_2_RR. **b** The cross-section of back-illuminated Si photoanode and the GDE cathode (figure not to scale for clarity).

To realize a PEC flow cell for CO_2_RR, a Si photoelectrode integrated flow cell was proposed for CO_2_RR (Fig. 1a). The left sidewall of the flow cell is the Si compartment, where water oxidation reaction (OER) takes place. Incident light was irradiated from the chamber with the open-hole plate, allowing the Si to absorb the light directly. The surface of Si was textured to micro-pyramid (Fig. S1) to decrease the surface reflectance (Fig. S2). The GDE was situated on the opposite of the Si compartment, to facilitate CO_2_RR. Various catalysts can be deposited onto the GDE for different kinds of CO_2_ reduction products.

To construct a decoupled Si photoanode in which its illumination and catalytic sites are located on different sides and enable light absorption from the back side (Fig. 1b), amorphous Si was deposited as passivation layer to saturate the dangling bonds on Si surface due to its superior chemical passivation effect (prepared by plasma enhanced chemical vapor deposition (PECVD), details in Methods)^28^. The amorphous Si passivated the Si substrate, exhibiting an ultra-high minority carriers lifetime (3275 μs) (Fig. S3), and long minority carrier diffusion length of 2015 μm (calculation details in the Methods). This ultra-long minority carrier diffusion length is much longer than the thickness of Si wafer (155 μm), enabling photogenerated minority carriers (holes) to move from one side of Si electrode to other side before recombining (Fig. 1a). Thus, light absorption and surface reaction can be decoupled by having the light illumination side and OER catalyst layer on be located on different sides of Si substrate. Furthermore, two doped thin films (p type and n type amorphous Si) were deposited on amorphous Si (prepared by PECVD, details in Methods) to construct a built-in electric field to enhance the driving force for photogenerated carriers (Fig. S4).

**Fig. 2:**
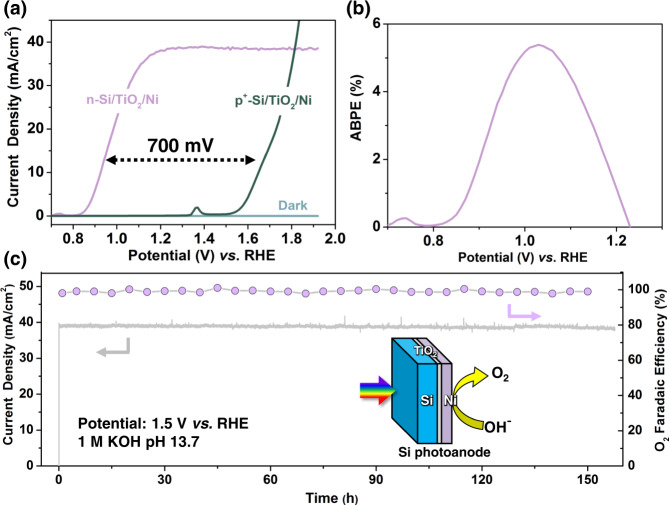
Performance of back-illuminated Si photoanode. **a** The current-voltage curves, **b** ABPE and **c** Time-dependent photocurrents (left-axis) and Faradaic efficiency toward O_2_ (right-axis) under simulated AM 1.5 G illumination. Inset: Schematic of the light absorption and reaction decoupled Si photoanode.

To evaluate the performance and stability of back-illuminated Si photoanode for water oxidation. 10 nm TiO_2_ protective layer was deposited by atomic layer deposition (ALD). The thickness of TiO_2_ was optimized to 10 nm in our previous work^40^, as the thickness presents no significant effect on the stability of the silicon photoanode for water oxidation when the thickness exceeds 8 nm^41^. Then 5 nm Ni with a larger surface-active area compared to thick Ni thin films deposited by direct current (DC) sputtering on TiO_2_ as OER catalyst for Si photoanodes (photograph of Si photoanode in Fig. S5). The back-illuminated Si photoanodes exhibit a dense surface morphology after TiO_2_ and Ni deposition (Fig. S7). For PEC measurements, the Si photoanode was evaluated under AM 1.5 G illumination (100 mW/cm^2^). To be consistent, 1 M KOH (pH 13.7) was used as the CO_2_RR in flow cell electrolyte^42^. The Si photoanode showed an early onset potential of 0.8 V vs. reversible hydrogen electrode (RHE) and high saturation photocurrent density (38 mA/cm^2^) (Fig. 2a), a significantly reduced onset potential compared with electrochemical OER. This photocurrent density is also much higher than that of wide band-gap photoelectrodes (smaller than 5 mA/cm^2^), demonstrating the merits of narrow band-gap photoelectrode for CO_2_RR in flow cells. The photogenerated voltage of this back-illuminated Si photoanode, determined by the difference in onset potential between the Si photoanode and degenerated p^+^-Si/TiO_2_/Ni electrode with no photo response (Fig. 2a), was about 700 mV at current density comprised between 1 and 38 mA/cm^2^, as indicated. Holes were the majority and minority carriers of the p^+^-Si/TiO_2_/Ni dark electrodes, respectively. To fairly compare surface reactions between the Si dark electrode and photoelectrode, the TiO_2_ protective layer and Ni catalyst layer of dark electrodes were prepared under same batch as Si photoanode (Fig. S8). A high applied bias photon-to-current efficiency (ABPE) of 5.4% was obtained (Fig. 2b). The high ABPE and stability can be attributed to the efficient light utilization efficiency achieved by the back-illumination structure. Furthermore, the stability of Si photoanodes was evaluated by a chronoamperometry test at a fixed applied potential of 1.5 V vs. RHE. The photocurrent remains stable for over 150 h (Fig. 2c). The Faradaic efficiency of O_2_ generation for Si photoanode is higher than 97% at 1.5 V vs. RHE under AM 1.5 G irradiation over a 150-h, indicating that most of the photogenerated holes are consumed for OER (Fig. 2c). The surface morphology is nearly unchanged after the stability test (Fig. S7). Moreover, grazing incidence X-ray diffraction (GIXRD) patterns of Si photoanode showed similar ITO diffraction peak intensity before and after stability (Fig. S9). Ni catalyst layer was stabilized on the surface of TiO_2_ by metal–support interaction^43^, and partially transformed to NiO_*x*_ or NiOOH after stability test (Fig. S6). This robust stability is attributed to the ALD-deposited TiO_2_ separating the electrolyte and Si photoanode, preventing corrosion. The superior activity and stability of this back-illuminated Si photoanode configuration have the potential to operate in flow cells with reduced cell voltage and improved energy utilization efficiency for CO_2_RR.

### High-rate CO_2_ reduction in PEC flow cell with controllable product distribution

**Fig. 3:**
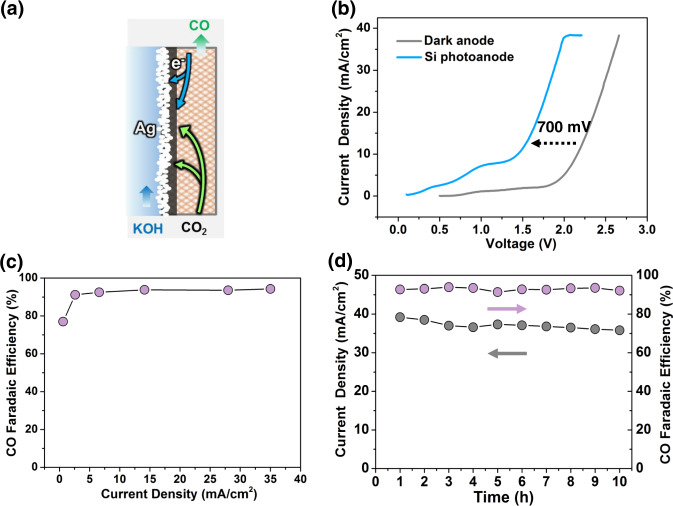
Performance of CO_2_ to CO with Si photoanode assistance. **a** The schematic of Ag cathode. **b** The *J*–*V* curves of CO_2_RR with and without Si photoanode in a two-electrode configuration. **c** The FE of CO at different current densities. **d** Time-dependent photocurrents (left-axis) and FE toward CO (right-axis) under simulated AM 1.5 G illumination.

Benefiting from the back-illuminated configuration, this Si photoelectrode could be used as the photoanode for OER in the CO_2_RR flow cell. The CO products were generated using metallic Ag as the catalyst (photograph in Fig. S10a) alongside our back-illuminated Si photoanode in flow cell with (photograph in Figs. S11, S12). 50 nm Ag catalyst was deposited on carbon paper by DC sputtering (Fig. 3a), which appeared to a continuous surface (Fig. S13). Ag catalyst is highly crystalline and shows a face-centered cubic crystal structure (Fig. S14). The CO_2_RR was carried out in 1 M KOH to suppress the hydrogen evolution, and the Si photoanode was performed under AM 1.5 G illumination (100 mW/cm^2^). To verify the benefits of back-illuminated Si photoanode in lowering cell voltage, the current–voltage (*J*–*V*) curves of CO_2_RR were collected in a two-electrode configuration (Fig. 3b). The back-illuminated Si photoanode promoted flow cell only required cell voltages as low as 1.44 V to obtain the current density of 10 mA/cm^2^, which is 720 mV lower than the conventional dark anode (p^+^-Si/TiO_2_/Ni) using Ni as the catalyst (2.16 V). This is consistent with the photovoltage of the back-illuminated Si photoanode (Fig. 2a). Therefore, the external energy input for CO_2_RR was significantly decreased by the reduction OER energy consumption. The performance of the back-illuminated Si photoanode-assisted CO_2_RR to produce CO was evaluated over Ag catalysts at different current densities (Fig. 3c), where the exclusive formation of gaseous CO and H_2_ is observed in the gas phase. The Ag catalyst exhibited a selectivity of more than 90% for the reduction of CO_2_ to CO under wide current density range of 3–38 mA/cm^2^ (Fig. 3c), which is consistent with previously reported results in electrochemical CO_2_ reduction^44–46^. Moreover, the durability of PEC flow cell for CO production was evaluated, where the FE of CO remained nearly unchanged throughout the test duration (Fig. 3d). To further evaluate the stability of this Si electrode-promoted flow cell, p^+^-Si/TiO_2_/Ni was used as dark electrode (Fig. S8). The current density of dark electrode continuously decreased during the 10 h stability test, having the same trend of decline as the Si photoelectrodes (Fig. S17). *J*–*V* curves of p^+^-Si/TiO_2_/Ni dark electrodes show no degradation during its 10 h stability test (Fig. S16). However, the formation of carbonate on the GDE surface (photograph in Fig. 15) was shown to inhibit CO_2_ transport and reduce the number of available catalytic sites after a 12 h stability test in dark (Fig. S17), leading to a decreased current density (Fig. 2c)^47,48^. Furthermore, carbonation of the electrolyte tends to shift the pH towards the neutral regime (from 13.7 to 13.4), resulting in higher solution resistance and unfavorable kinetics for OER, consistent with former investigations^49,50^. Therefore, the increased cell voltage can be attributed to the formation of carbonate on the GDE and the decreased pH of electrolyte.

**Fig. 4:**
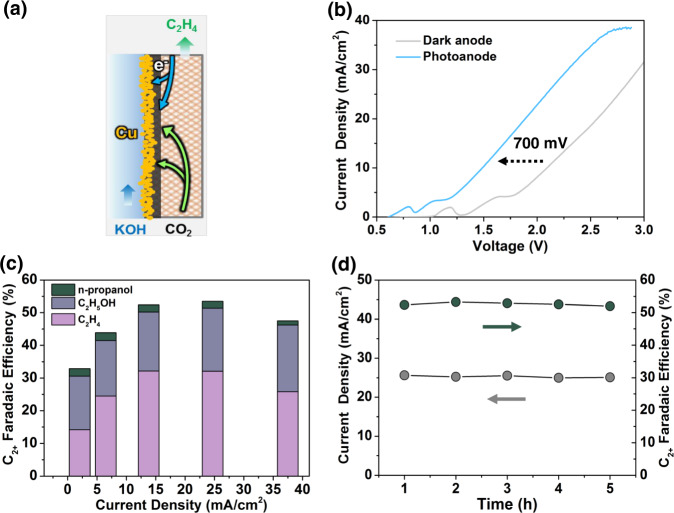
Performance of CO_2_ to C_2+_ with Si photoanode assistance. **a** The schematic of Cu cathode. **b** The *J*–*V* curves of CO_2_RR with and without Si photoanode in a two-electrode configuration. **c** The FE of C_2+_ at different potentials. **d** Time-dependent photocurrents (left-axis) and FE toward C_2+_ (right-axis) under simulated AM 1.5 G illumination.

In addition to CO, multi-carbon products with high energy densities such as C_2_H_4_ and C_2_H_5_OH are also producible from this PEC flow cell configuration^20,51^. 100 nm Cu was sputtered on carbon paper GDE in this work (photograph in Fig. S10b). X-ray diffraction and X-ray photoelectron spectroscopy confirmed the existence of metallic Cu (Figs. S18, S19). The Cu catalyst was combined with the back-illuminated Si photoanode to carry out the CO_2_RR in flow cell using 1 M KOH (pH 13.7) as the electrolyte (Fig. 4a) (photograph in Fig. S20).

Compared to the system without back-illuminated Si photoanodes, external the voltage input was lower by 700 mV in Si photoanode-assisted flow cell, indicating that the high photovoltage can be provided by the back-illuminated Si photoanode to produce multi-carbon products (Fig. 4b). The FE for C_2+_ products including C_2_H_4_, C_2_H_5_OH, and propanol reaches 53% on Cu catalysts, comparable to FE of unmodified metallic copper electrocatalysts (Fig. 4c)^52–54^. Furthermore, the FE of C_2+_ products reached 53% with the help of Si photoanode during its 5 h operation (Fig. 4d). Therefore, the back-illuminated Si photoanode can feasibly be coupled with CO_2_RR flow cell operating at high reaction rates and maintain the same FE and stability as electrocatalytic CO_2_ reduction flow cells.

### Energy conversion of PEC flow cell with Si photoelectrode

The back-illuminated Si photoanode has been successfully coupled with the flow cell, and maintained a decent activity and stability for converting CO_2_ to CO, C_2_H_4_ and C_2_H_5_OH. The back-illuminated Si photoanode effectively reduced the energy consumed from the external electrical power supply. To assess the contribution of Si photoanode to the CO_2_ reduction system, the STF conversion efficiency of the Si promoted cell was calculated by Eq. (1):

1 $${\eta }_{{{{{{\rm{STF}}}}}}}=\sum \frac{({E}^{0}-{V}_{{{{{{\rm{II}}}}}}})\times {{{{{\rm{I}}}}}}\times {{{{{\rm{FE}}}}}}}{{P}_{{{{{{\rm{in}}}}}}}}\times 100\%$$

The thermodynamic potential of the respective product (*E*^0^) of CO, C_2_H_4_, C_2_H_5_OH, and C_3_H_7_OH are respectively equal to 1.334, 1.150, 1.147, and 1.131 V^20,51^. *V*_II_ is the cell voltage measured in two electrodes (Figs. 3b, 4b). *I* is the operating current density at different voltages. FE is Faradaic efficiency for target product. *P*_in_ is the input power (100 mW/cm^2^ in this work). The active area of the Si photoanode was 1 cm^2^.

**Fig. 5:**
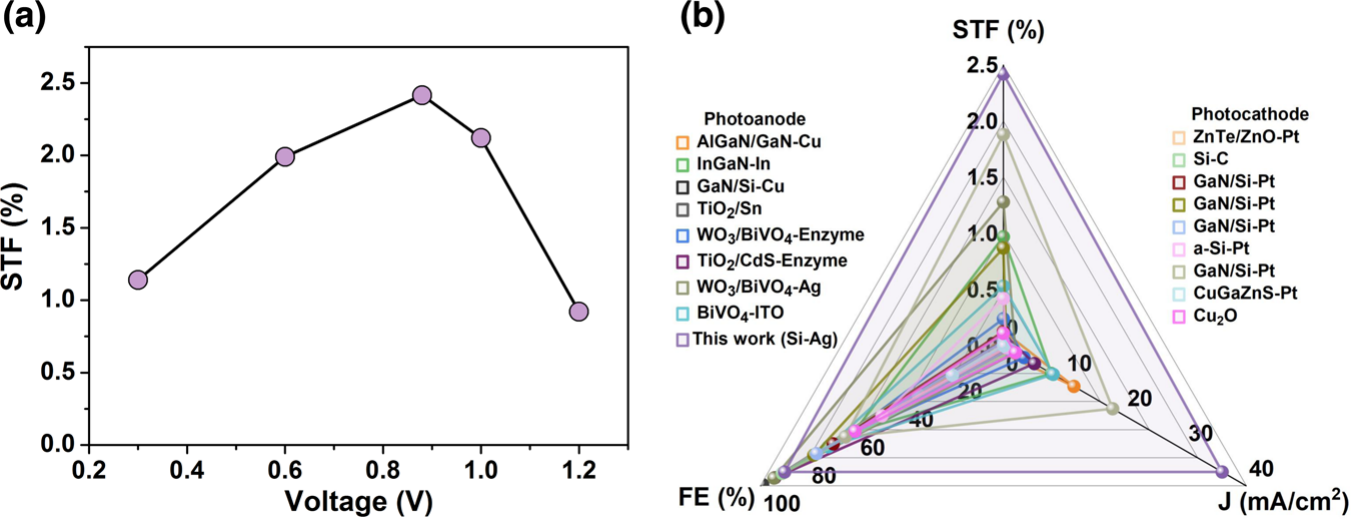
Calculation and comparison of STF efficiency. **a** STF efficiency of PEC Si photoanode for the conversion of CO_2_ to CO and **b** Performance comparison of photoanodes and photocathodes promoted cell for PEC CO_2_RR. Chart visualizing data on FE of products, photocurrent density, and STF for PEC CO_2_RR. Details are provided in Tables S1, S2.

For the conversion of CO_2_ to CO, the FE of CO was larger than 90% in the voltage range of 0.3–1.334 V (Fig. 3b), leading to a high STF efficiency of 2.42% (Fig. 5a). To the best of our knowledge, this is the highest STF efficiency among photoanodes assisted cell for PEC CO_2_RR (Fig. 5b), also surpassing the STF efficiency of state-of-the-art photocathodes for PEC CO_2_RR (Fig. 5b). The STF for C_2+_ products reaches 0.29% (Fig. S21), which is lower than that of CO because the generation of C_2_H_4_ and C_2_H_5_OH (8 and 12 electrons reactions, respectively) is kinetically more difficult than the two-electron reduction products (CO)^20,54^. Although the STF efficiency in this work is lower than photoelectrodes fabricated from multi-junction III–V semiconductors under concentrated illuminations, the Si-based PEC flow cell holds the merit of low costs and facile fabrication processes^18,32^. Therefore, the Si photoanode could effectively reduce the high overpotential in OER, while maintaining a reasonable performance to produce CO_2_ reduction to CO or C_2+_ products compared with the conventional electrochemical CO_2_ reduction in the flow cell.

## Discussion

In summary, this paper demonstrates a PEC flow cell for CO_2_ reduction with a back-illuminated Si photoanode that improves energy utilization efficiency. To overcome the contradiction between the opaque semiconductor light absorber and gas diffusion electrode in a flow cell, the Si photoelectrode was passivated by amorphous Si to obtain an ultra-long minority carrier diffusion length and separate the light absorption region from the catalytic sites. This decoupled structure enables the light to be introduced from the anode compartment side instead of the cathode side, when the photoanode is used as a sidewall of the cell. In this configuration, a high reaction rate (38 mA/cm^2^) for CO_2_ reduction was achieved in the PEC flow cell for CO_2_RR. For the conversion of CO_2_, the back-illuminated Si photoanode-assisted PEC flow cell shows an STF efficiency of 2.42% for CO with a FE of 90% using Ag catalyst. The direct PEC flow cell for CO_2_RR to C_2+_ products is achieved at a FE of 53% on Cu catalyst and an STF of 0.29% is achieved. The design strategy in this work opens promising approaches based on Si photoelectrodes to generate solar fuels from CO_2_ and H_2_O towards industrial application.

## Methods

### Fabrication of Si photoelectrode with amorphous Si passivation layer

Czochralski (CZ) c-Si wafers (n-type) with the resistivity of 1–5 Ω cm, 155 μm thickness and (100)-orientation were used as the substrate for Si electrode peparation. Silicon wafer was first textured using a mixture of potassium hydroxide (KOH) and isopropyl alcohol (IPA) (Top view SEM images in Fig. S1), then cleaned by standard RCA cleaning methods and finally dipped in 5% HF for 60 s to get rid of surface native oxide thin film. The Si wafer was then put into the PECVD chamber. 5 nm Intrinsic amorphous Si thin films were used to passivate the front and back side surfaces of Si substrate. n^+^ amorphous Si and p^+^ amorphous Si were deposited on the intrinsic amorphous Si (Fig. S4). WCT-120 lifetime tester from Sinton Instruments was applied for minority carrier lifetime test.

### Deposition of TiO_2_ protective layer

10 nm of amorphous TiO_2_ was deposited onto the above Si photoelectrodes at 150 °C in a custom-made ALD system, using Titanium (IV) isopropoxide (TTIP, Sigma-Aldrich, ≥99.9999%) and ultrapure water as precursors. The precursors were held at 70 °C and 25 °C, respectively. One ALD cycle consists of TTIP dose for 1 s, N_2_ purge for 10 s, water dose for 0.1 s and N_2_ purge for 10 s. The growth per cycle for TiO_2_ is 0.3 Å/cycle.

### Deposition of Ni catalytic layer

Ni was deposited by custom-made direct current magnetron sputtering. The sputtering target is Ni with high purity (99.99%). The chamber base pressure was kept at 10^−5^ Pa, and then high-purity Ar flow was fed into the chamber. The gas flow was controlled at 20 sccm by mass flow controllers. The process pressure was fixed at 1 Pa and deposition time can be regulated to adjust the thickness of the film.

### Preparation of dark Si electrode

Czochralski (CZ) p-type c-Si wafers with the resistivity of 0.001–0.005 Ω cm, 525 μm thickness and (100)-orientation were used as substrate. The ohmic back contacts were formed by rubbing the back side surfaces of the Si samples with a Ga–In alloy (99.9% metal basis, Alfa Aesar).

### PEC measurements

PEC measurements were performed using a three-electrode configuration with Si electrode as the working electrode, Hg/HgO as the reference electrode in alkaline electrolyte. Schematic illumination and photograph of the apparatus used in the PEC measurement are shown in Fig. S22. The potentials were converted into values against reversible hydrogen electrode using the Nernst Eq. (2) for dark cathode and photoanodes.

2 $${V}_{{{{{{\rm{RHE}}}}}}}={V}_{{{{{{\rm{Hg}}}}}}/{{{{{\rm{HgO}}}}}}}+0.098(V)+0.059{{{{{\rm{pH}}}}}}$$

The potentiostat (CompactStat.e20250, IVIUM) was used to measure the *J*–*V* curves and chronoamperometry (without iR compensation). *J*–*V* curves of samples were measured with a scan rate of 50 mV s^−1^ under the irradiation of a 150 W xenon lamp (SAN-EI ELECTRIC CO., LTD) equipped with an AM 1.5 G filter. The active geometric areas of the working electrode were calibrated by the software Image J.

3 $${{{{{\rm{ABPEs}}}}}}=\frac{{{{{{\rm{I}}}}}}\times (1.23-{V}_{{{{{{\rm{b}}}}}}})}{P}\times 100\%$$

The applied bias photon-to-current efficiencies of photoanode were calculated using the *J*–*V* curves with an assumption of 100% Faradaic efficiency, according to Eq. (3). Where *I* (mA/cm^2^) is the photocurrent density under an applied bias of *V*_b_ (V vs. RHE), and *P* is the incident illumination intensity (mW/cm^2^) (100 mW/cm^2^ in this work).

### Analysis of CO_2_ reduction products

During electrolysis, the gas mass flow controller was set to 20 sccm, the peristaltic pump was set to 30 rpm (photograph in Fig. S23), gas products were quantified using an online gas chromatography system (GC7890B, Agilent Technologies, Inc.). H_2_, O_2_, and N_2_ were detected by thermal conductivity detector (TCD) (MolSieve 5A packed column, Agilent Technologies, Inc.) and CO was detected by back-flame ionization detector (FID) (Porapak Q packed column, Agilent Technologies, Inc.). A methanizer was installed to enable the back FID to detect CO with 1000 times higher sensitivity. Ar was used as the carrier gas. After passing through the reactor, the gas was allowed to flow directly into the gas sampling loop of the gas chromatography for online gaseous product analysis.

The Faradaic efficiencies of the gas products were calculated by using the concentrations (ppm) detected by the GC as following Eq. (4):

4 $${{{{{\rm{FE}}}}}}\%=\frac{{{{{{\rm{ppm}}}}}}\times {{{{{\rm{flow}}}}}}\,{{{{{\rm{rate}}}}}} \, ({{{{{\rm{sccm}}}}}})\times \frac{{{{{{{\rm{nFp}}}}}}}_{0}}{RT}}{{j}_{{{{{{\rm{total}}}}}}} \, ({{{{{{\rm{mAcm}}}}}}}^{\mbox{-}2})}\times 100\%$$

Where *n* is the number of electrons transferred to CO_2_ to produce a given product (*n*(CO) = 2; *n*(C_2_H_4_) = 12; *n*(C_2_H_5_OH) = 12), *p*_0_ is the pressure (101.325 kPa) and *T* = 273.15 K, *R* is the gas constant (8.314 J mol^–1^ K^–1^).

### Characterization

The thickness of amorphous Si, TiO_2_, Ni, Au and Ag layers on polished Si(100) monitor substrate was determined by spectroscopic ellipsometer (M-2000 DI, J.A. Woollam Co., Inc.) at 60° and 70° incident angle, by fitting the amplitude ratio (*Ψ*) and phase shift (*Δ*) of polarized light with the Cauchy dispersion model for a-Si and TiO_2_, and a tabulated metallic model for Ni, Au, and Ag. The morphology was carried out by field emission scanning electron microscope (FESEM, Hitachi S-4800, 5 kV). XPS analysis was conducted on a Physical Electronics PHI 1600 ESCA system with an Al Kα X-ray source (1486.6 eV). The binding energy was calibrated against the C 1 s photoelectron peak at 284.6 eV as the reference. The optical reflectance measurement of the Si was performed using spectrophotometer (Shimadzu UV-3600). The X-ray diffraction (XRD) (D/MAX-2500, Rigaku) spectra were collected over a 2*θ* range from 20° to 80° at a scanning speed of 0.02° per step.

### Calculation of the minority carrier diffusion length

5 $$\frac{1}{{{{{{{\rm{\tau }}}}}}}_{{{{{{\rm{eff}}}}}}}}=\frac{1}{{{{{{{\rm{\tau }}}}}}}_{{{{{{\rm{bulk}}}}}}}}+\frac{2S}{W}$$

The surface recombination velocity can be calculated by Eq. (5) for the Si electrode with a-Si passivation, where *W* is the thickness of Si wafer, *τ*_bulk_ is the bulk minority carrier lifetime, *S* is the surface recombination velocity.

6 $${L}_{p}=\sqrt{{{{{{{\rm{\tau }}}}}}}_{p}{D}_{p}}$$

The minority carrier diffusion length of Si photoelectrode can be calculated by Eq. (6), where *τ*_p_ is the minority carrier lifetime of n-type Si, D_p_ is the diffusion coefficient of hole (*D*_p_ = 12.4 cm^2^/s, at 298 K)^55,56^.

## Supplementary information

Supplementary Information

## Data Availability

All data generated or analyzed during this study are included in the published article and its Supplementary Information. Source data are provided with this paper.

## Source data

Source Data
