# Supplementary material for: Back-illuminated photoelectrochemical flow cell for efficient CO2 reduction
Source: Nat Commun. 2022 Nov 19;13:7111. doi: 10.1038/s41467-022-34926-x (PMC9675791; doi:10.1038/s41467-022-34926-x)
Supplement: Supplementary file 1 — Supplementary Information [file 41467_2022_34926_MOESM1_ESM.pdf]

Supplementary Information

Back-illuminated Photoelectrochemical Flow Cell for Efficient CO_2 Reduction

Bin Liu[1,2,3,4,§], Tuo Wang[1,2,3,§], Shujie Wang[1,2,3], Gong Zhang[1,2,3], Dazhong Zhong[1,2,3], Tenghui Yuan[1,2,3], Hao Dong[1,2,3], Bo Wu[1,2,3] and Jinlong Gong[1,2,3,4]*

[1]*School of Chemical Engineering and Technology; Key Laboratory for Green Chemical Technology of Ministry of Education, Tianjin University; Tianjin 300072, China.*

[2]*Collaborative Innovation Center of Chemical Science and Engineering (Tianjin), Tianjin 300072, China.*

[3]*Haihe Laboratory of Sustainable Chemical Transformations, Tianjin 300192, China.*

[4]*Joint School of National University of Singapore and Tianjin University, International Campus of Tianjin University, Binhai New City, Fuzhou 350207, China.*

[§]These authors contributed equally to this work.

*Corresponding author. E-mail: jlgong@tju.edu.cn.

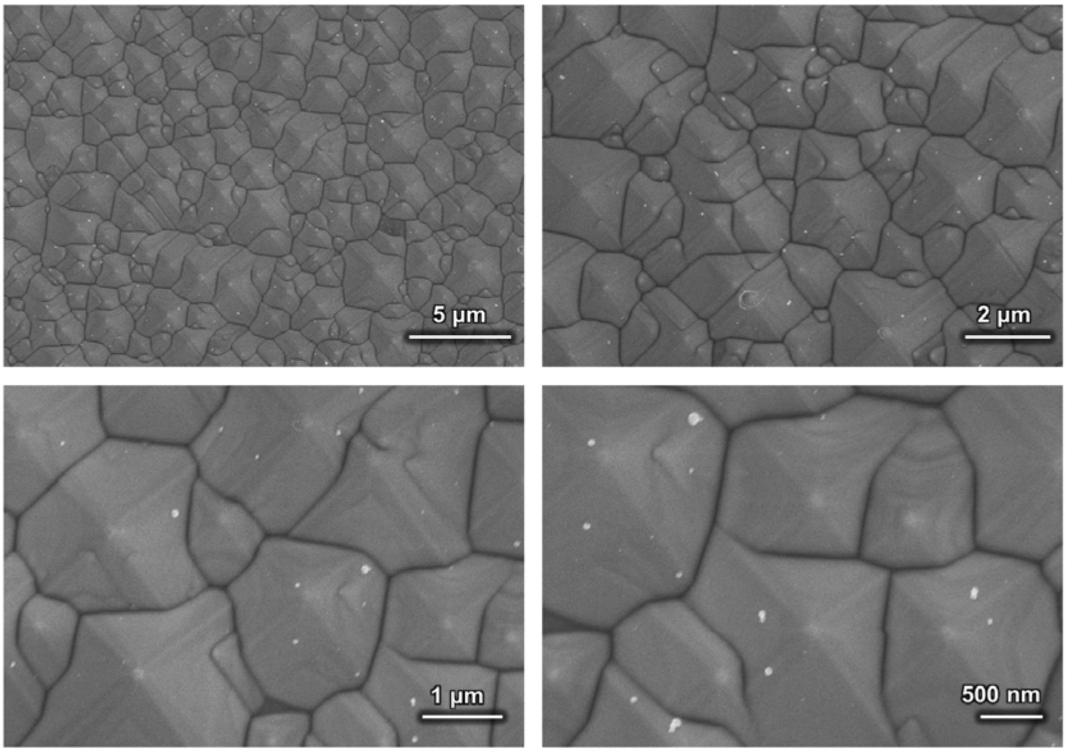

Figure S1 Top view SEM images of Si substrate after texture without TiO_2 and Ni layer.

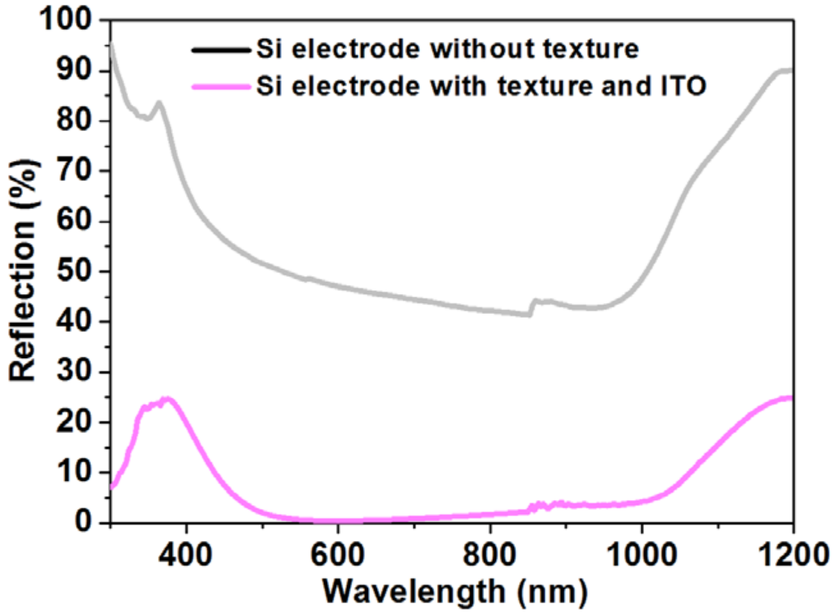

Figure S2 The total reflection spectra of Si photoanode with or without surface texture and ITO layer.

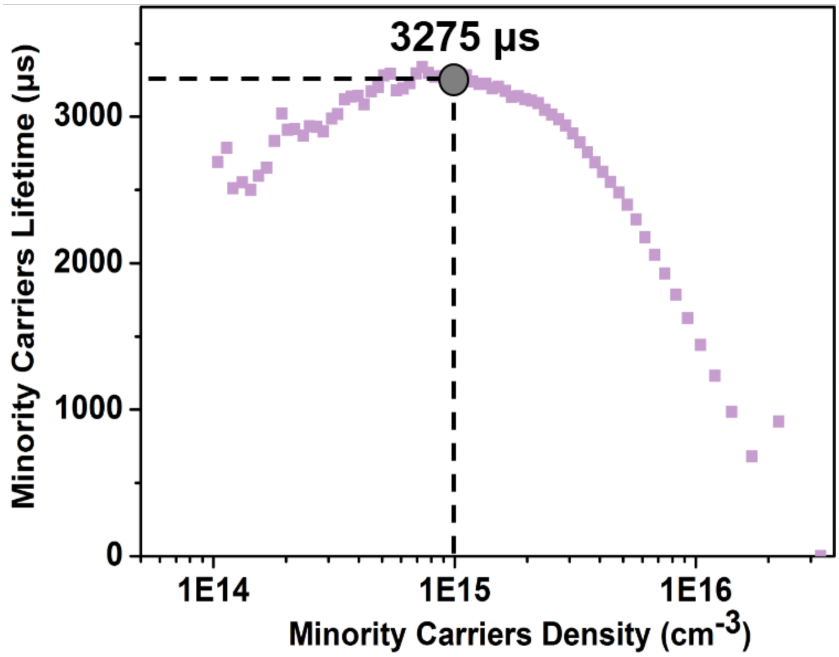

Figure S3 The minority carriers time of Si photoanode with a-Si passivation

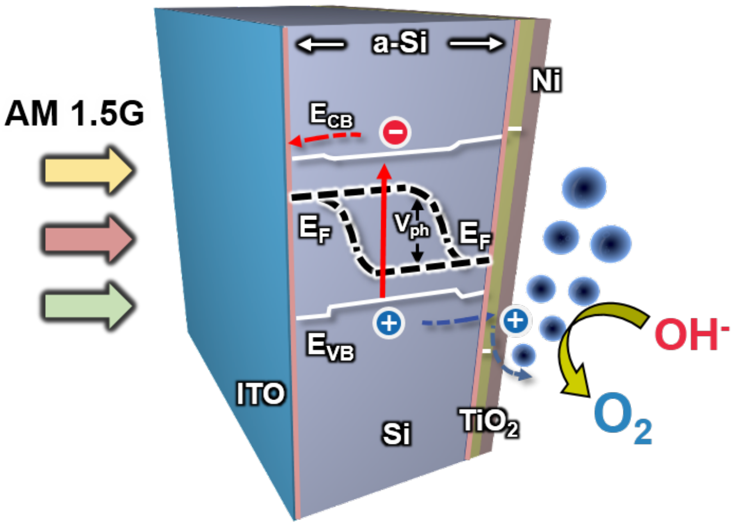

Figure S4 Schematic of the back-illuminated Si photoanode.
E_{CB} is the conduction band edge, E_{VB} is the valence band edge, and E_F is the quasi Fermi level under illumination. V_{ph} is the photovoltage generated by Si p-n heterojunctions. Then, the indium tin oxide (ITO) layers were deposited as anti-reflection layer and carrier collector.

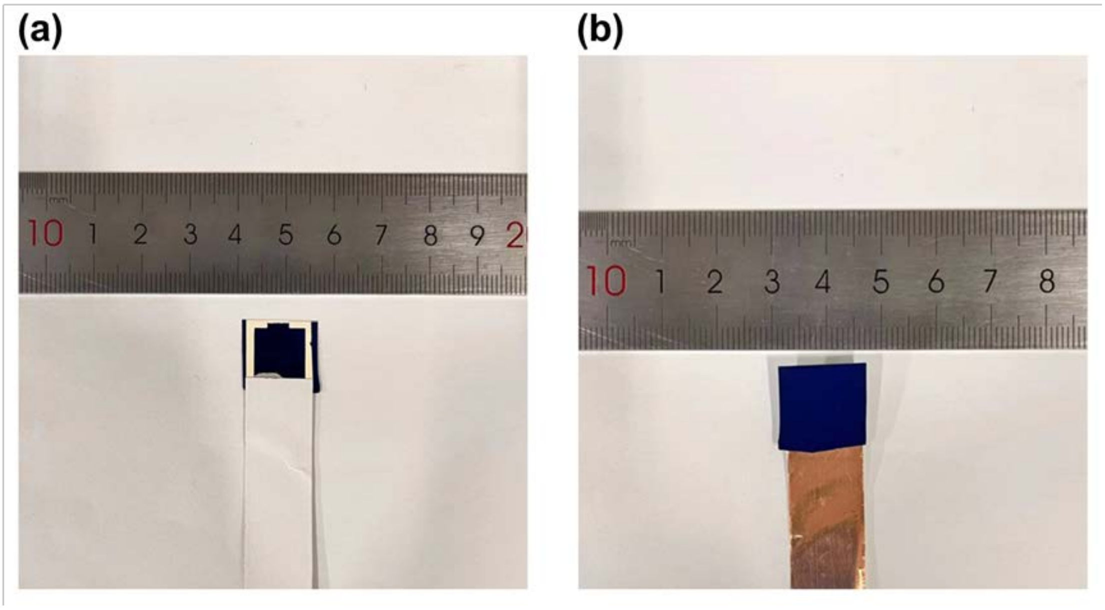

Figure S5 Photograph of back-illuminated Si photoanode. (a) light-facing side and (b) surface reaction side. The illumination area and surface reaction are both 1 cm^2.

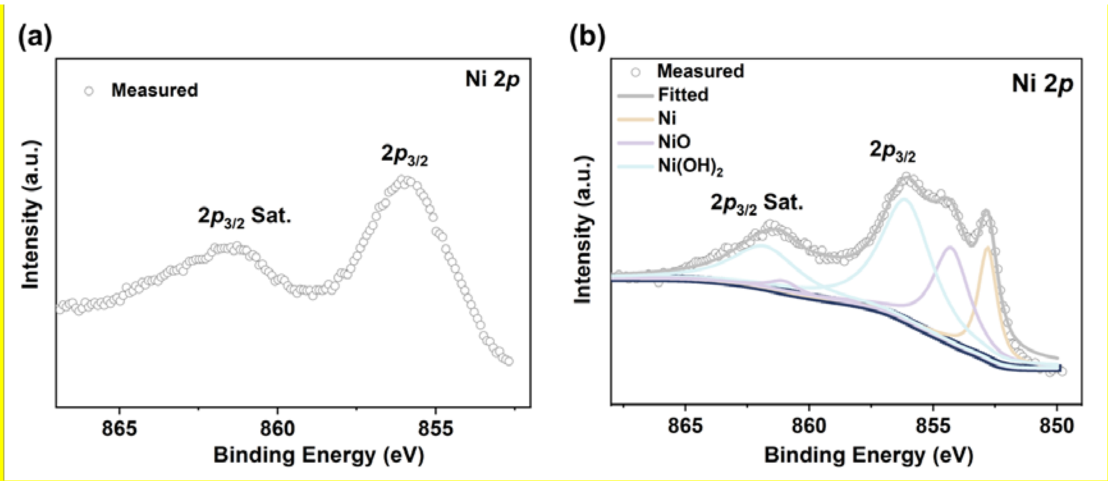

Figure S6 XPS of Si photoanode. (a) before and (b) after stability test.

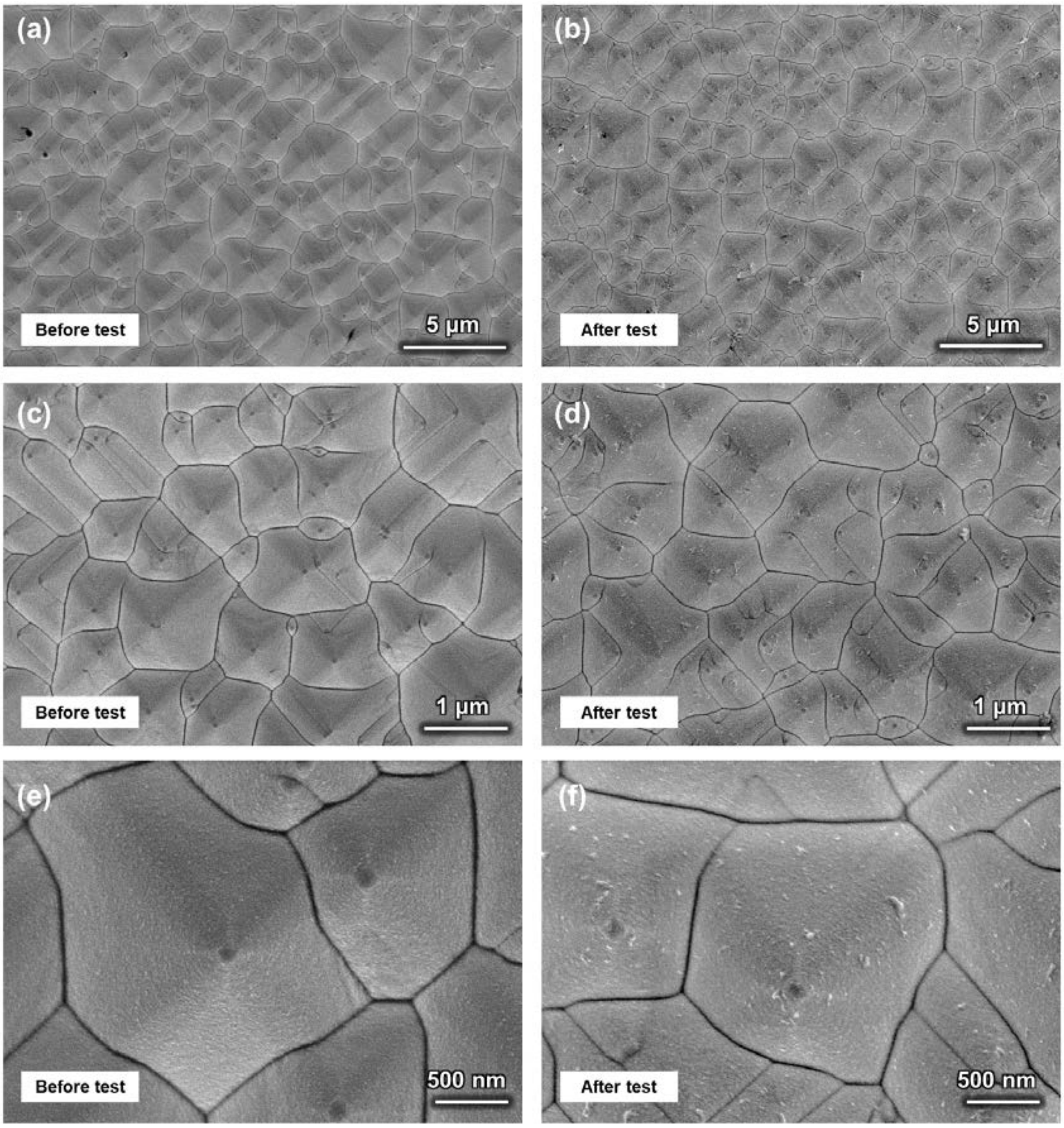

Figure S7 Top view SEM images of Si photoanode before (a, b, c) and after stability test (b, e, f).

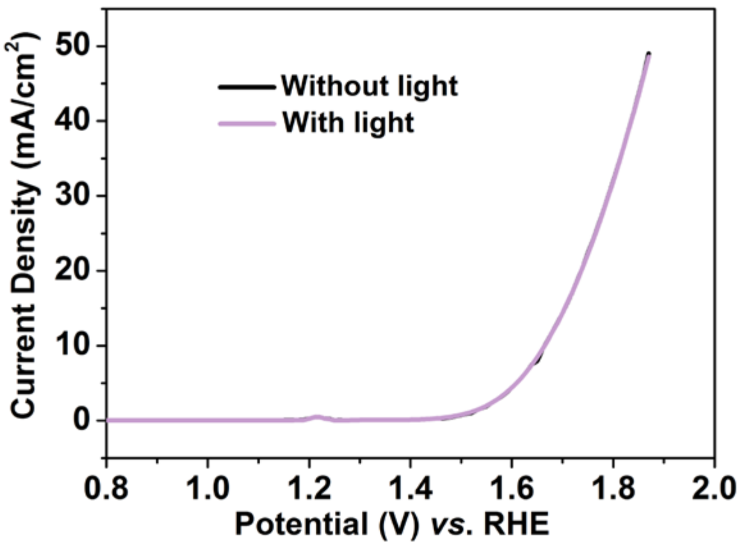

Figure S8 J-V curves of p$^+$-Si/TiO_2/Ni anode with and without light.

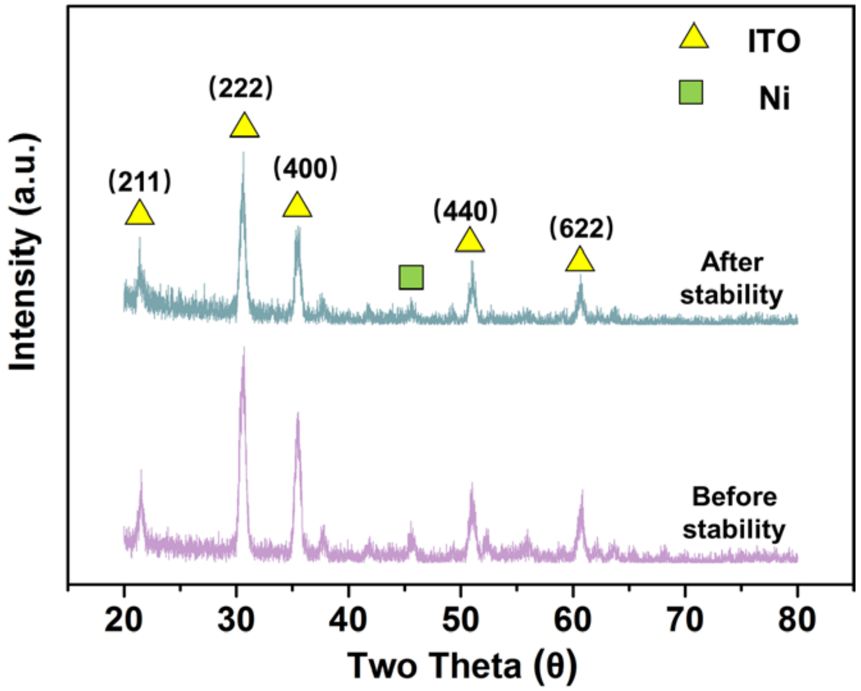

Figure S9 GIXRD patterns of Si photoanode (a) before and (b) after stability test.

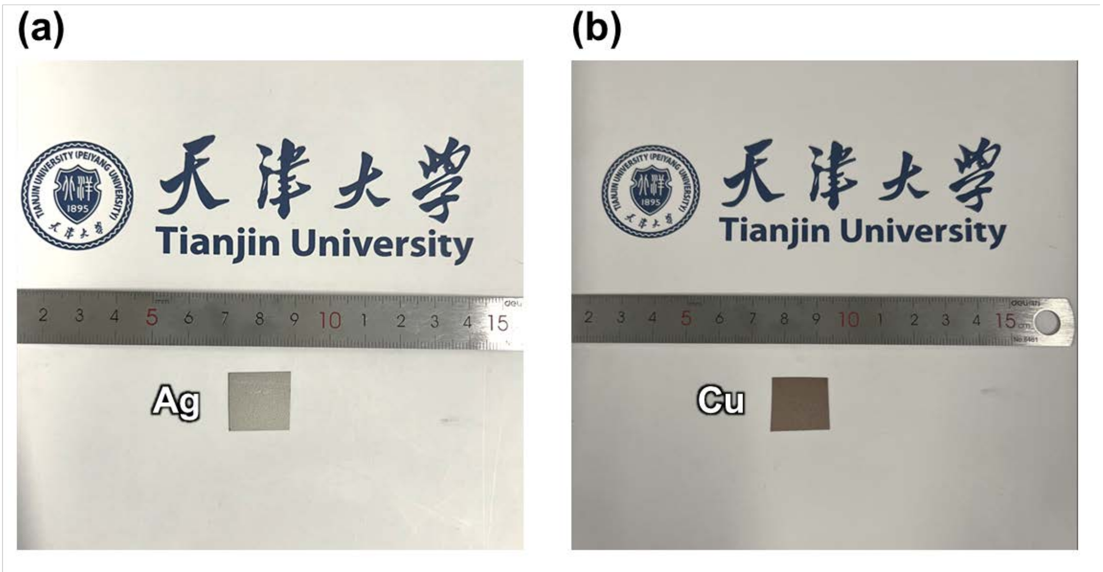

Figure S10 Photograph of (a) Ag and (b) (Cu) deposited gas diffusion electrode.

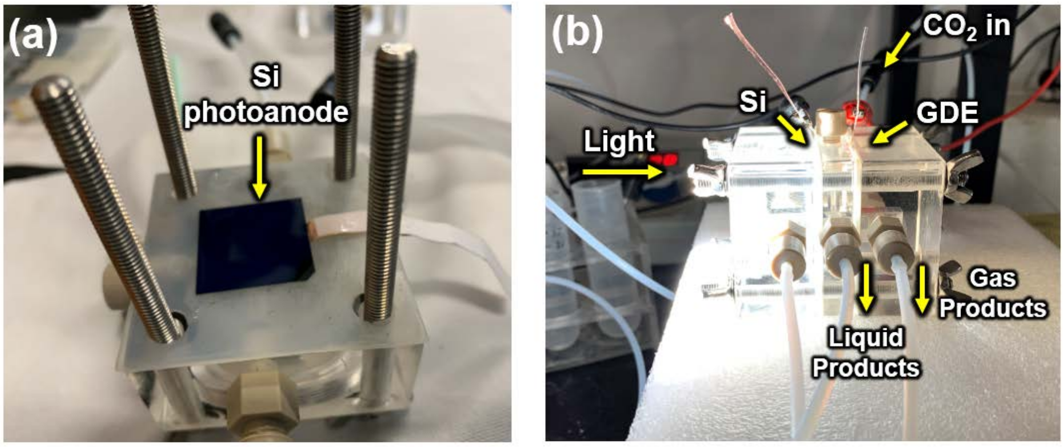

Figure S11 Photograph of (a) back-illuminated Si photoanode in flow reactor and (b) apparatus used in the PEC CO_2 reduction reaction measurement.

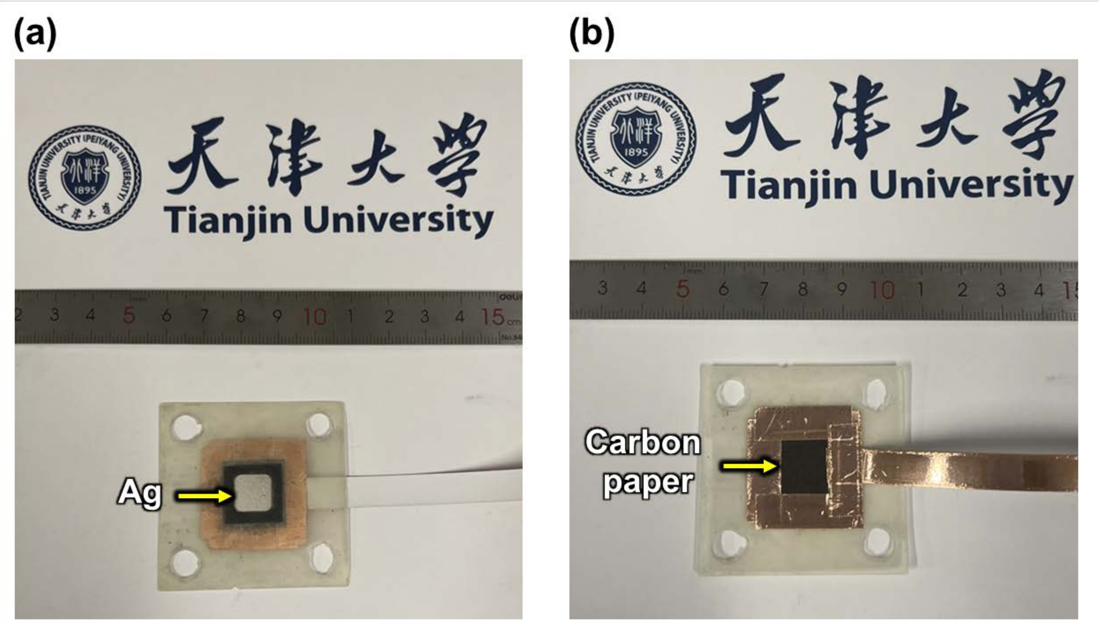

Figure S12 Photograph of (a) reaction side and CO_2 diffusion side of (b) Ag equipped rubber gasket. The reaction area is 1 cm^2.

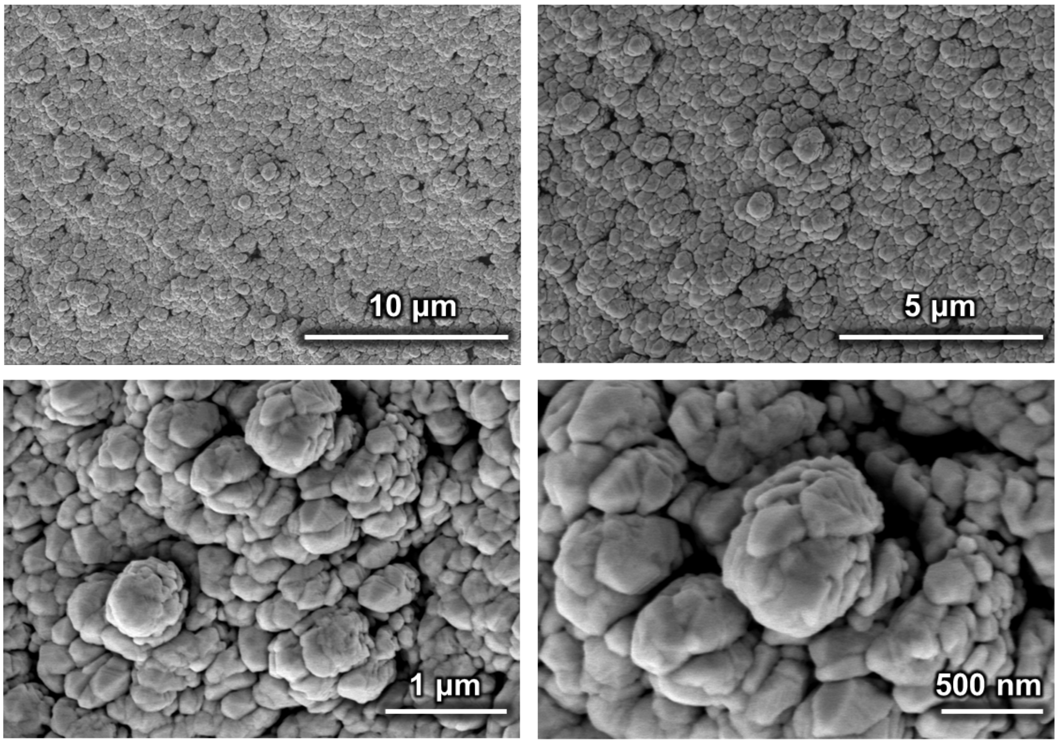

Figure S13 Top view SEM images of Ag catalyst.

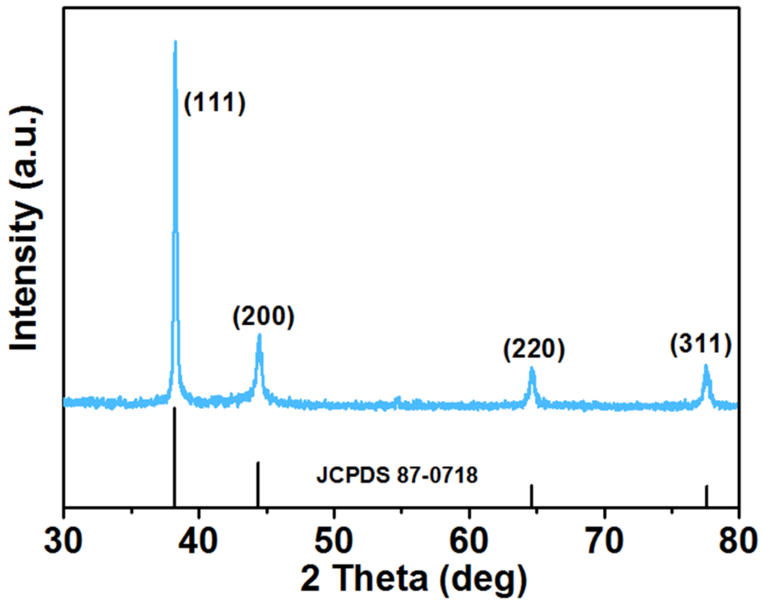

Figure S14 XRD pattern of Ag catalyst.

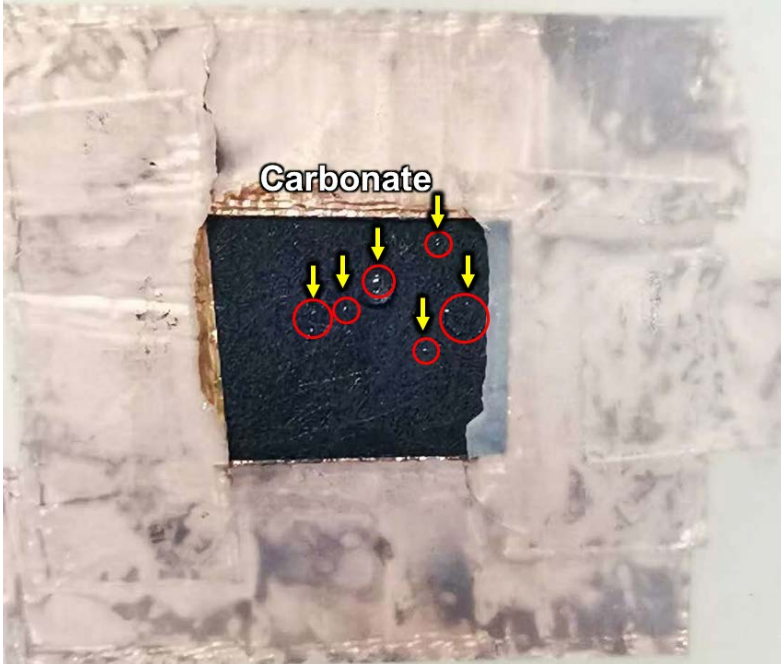

Figure S15 Photograph of the macroporous layer side of the GDE showing carbonate precipitation (circled in red) after continuous operation for 10 h.

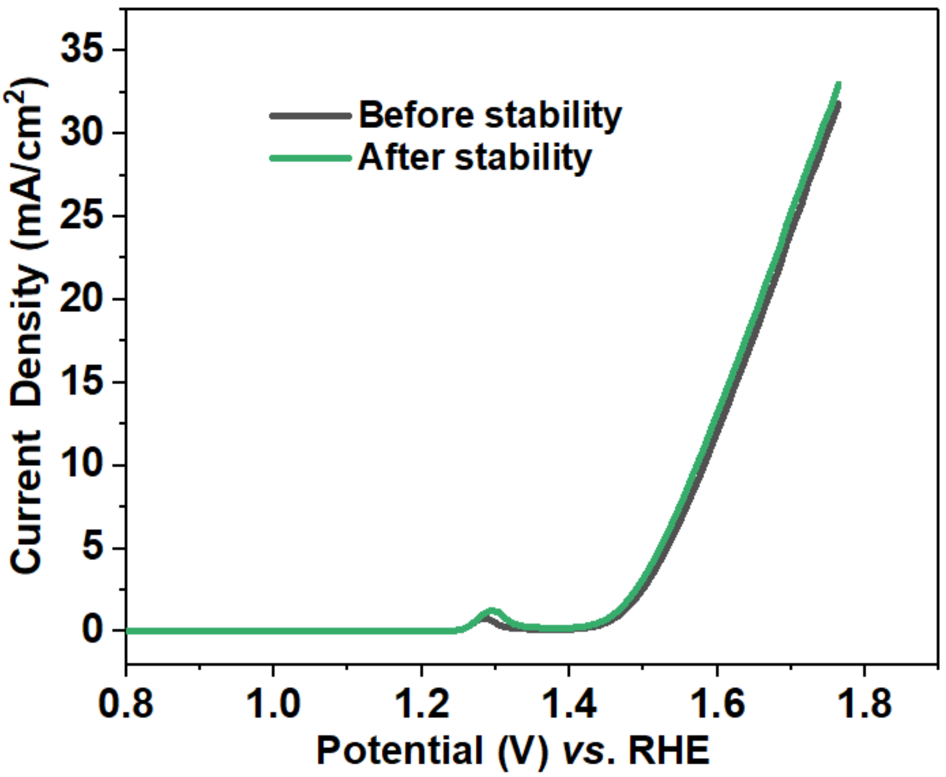

Figure S16 J-V curves of p^+-Si/TiO_2/Ni anode before and after stability test.

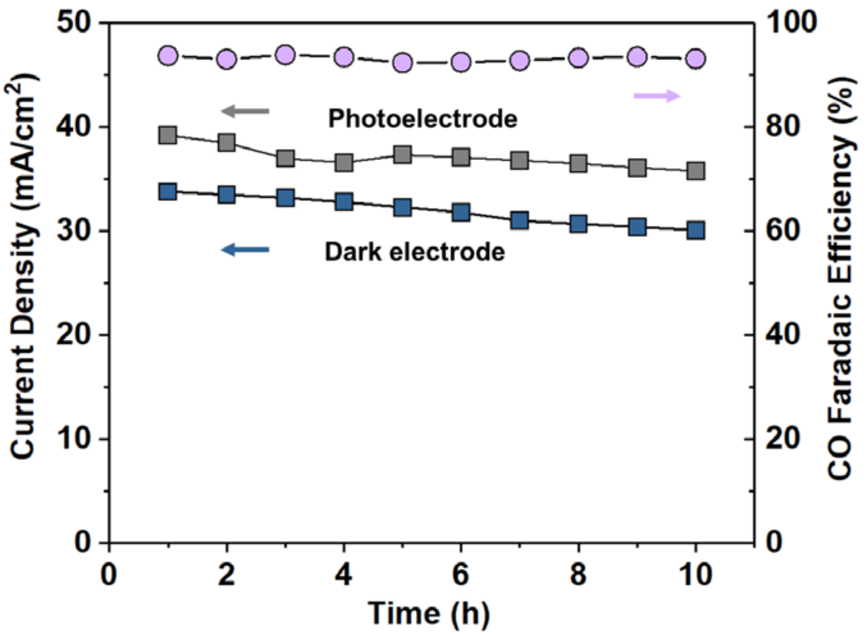

Figure S17 Stability comparison of dark anode (p$^+$-Si/TiO_2/Ni) and Si photoanode for CO_2RR in flow cell.

The non-photoactive electrode (p$^+$-Si/TiO_2/Ni) with the same TiO_2 and Ni as photoelectrode was used as anode to evaluate the stability under dark. The stability test set at constant 2.6 V in two-electrode configuration.

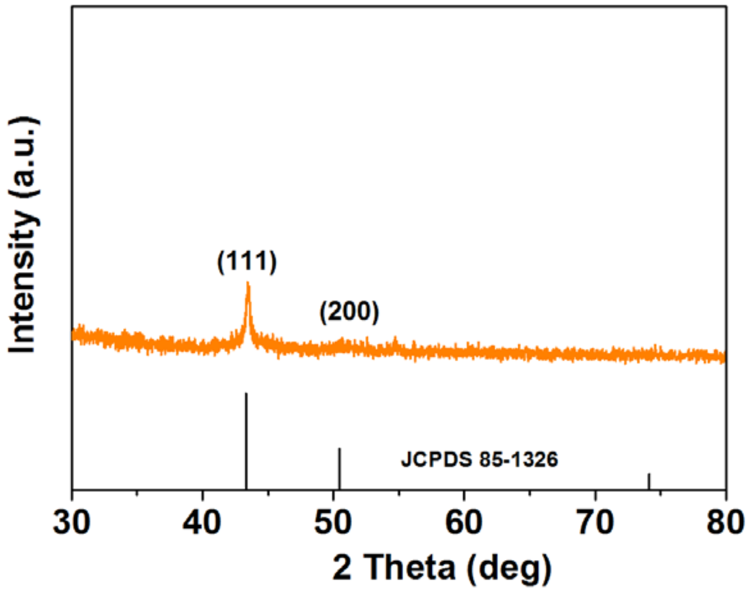

Figure S18 XRD pattern of Cu catalyst.

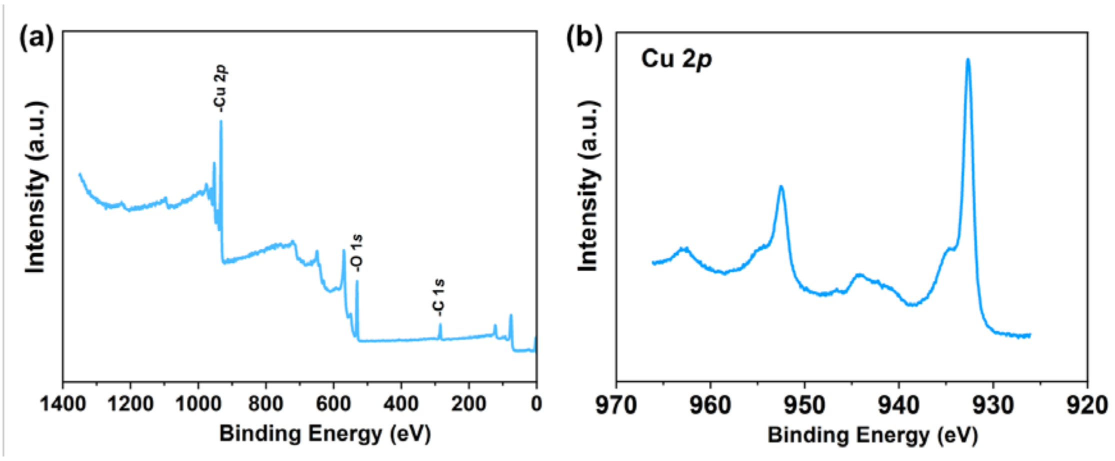

Figure S19 XPS of as-prepared Cu catalyst. (a) XPS survey spectrum. (b) XPS spectrum of Cu 2p.

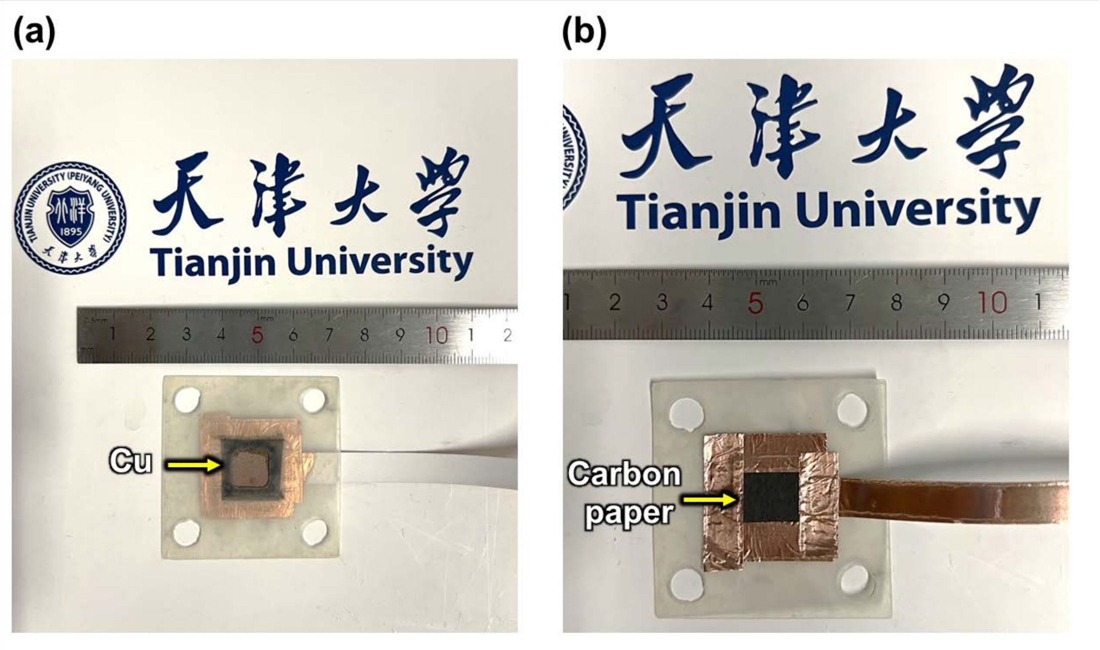

Figure S20 Photograph of (a) reaction side and (b) CO_2 diffusion side of Cu GDE equipped rubber gasket. The reaction area is 1 cm^2.

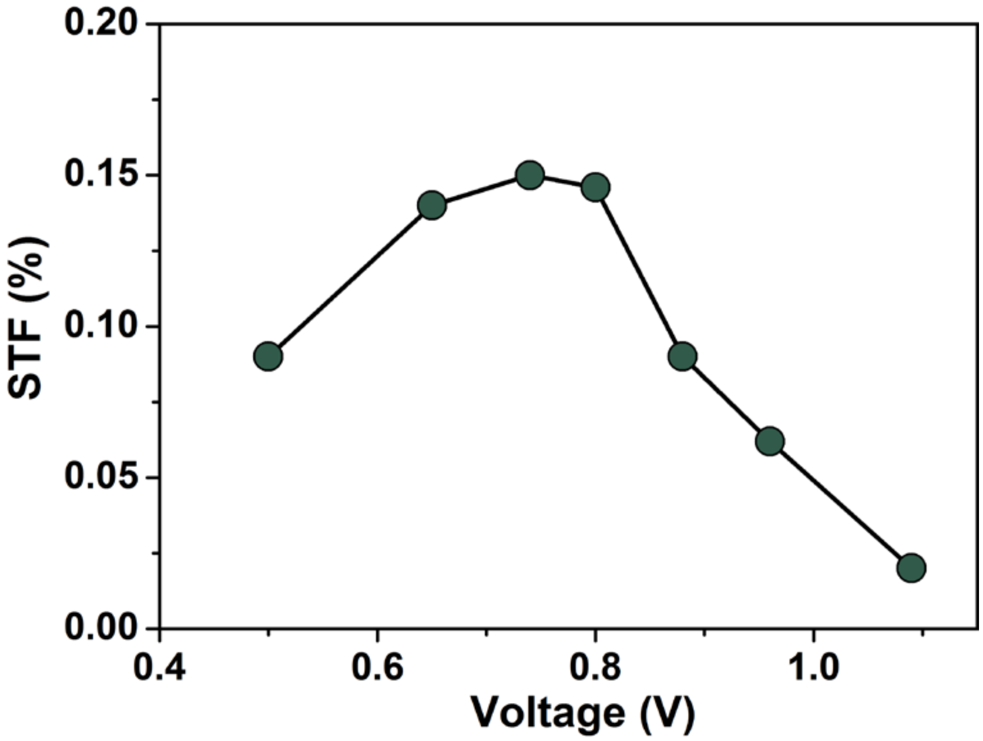

Figure S21 STF efficiency of PEC Si photoanode for the conversion of CO_2 to C_{2+}.

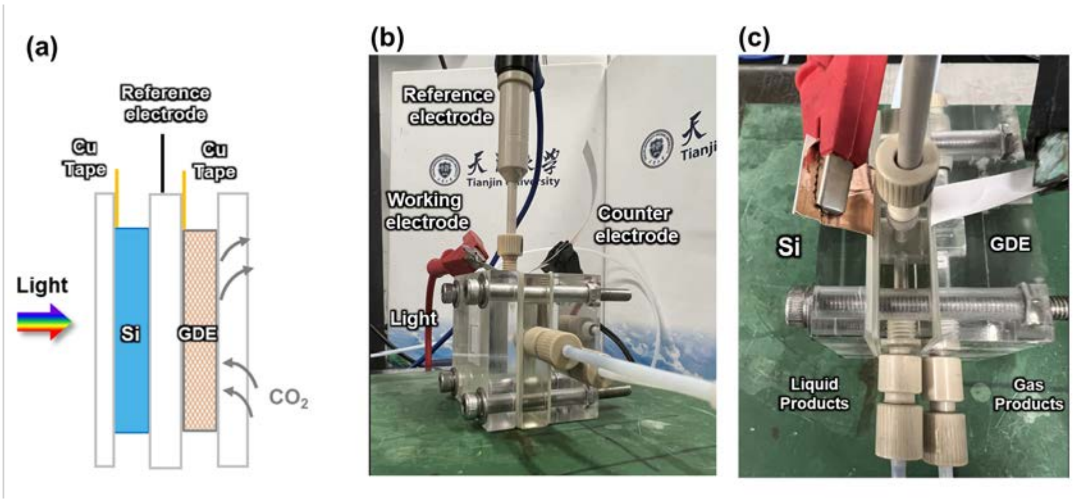

Figure S22 Schematic illustration (a) and photograph of (b) side- and (c) top-view of photograph of Si photoanode promoted flow cell in three-electrode configuration.

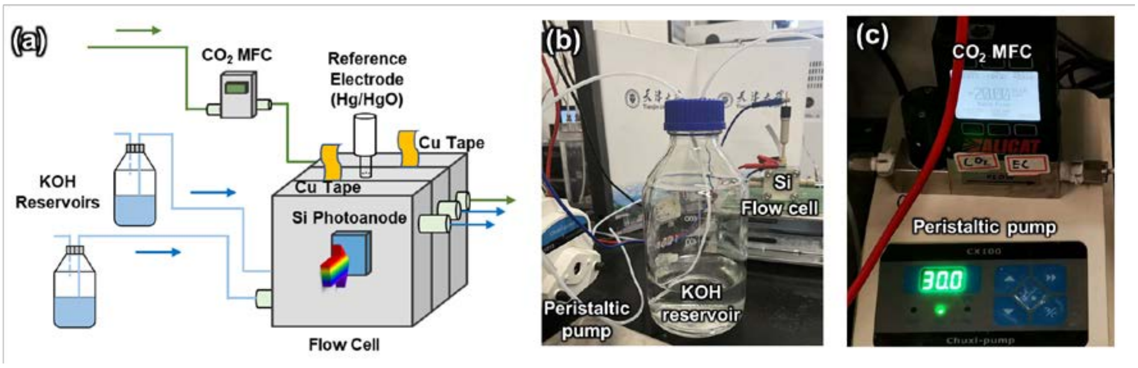

Figure S23 Schematic (a) and photographs of (b) the gas-fed, flowing liquid electrolyte experimental setup and (c) the CO_2 MFC and peristaltic pump.

Table S1 Performance comparison of photoanodes promoted CO_2RR

Photoanode	Cathode	Catalyst	Reduction products	FE (%)	Current Density (mA/cm^2)	Irradiation Conditions / Intensity	STF (%)	Reference
AlGaN/GaN	Cu	NiO	C_2H_4, C_2H_5OH	3	11.6	λ > 400 nm / 15 Suns	0.13	1
InGaN	In	NiO	HCOOH	61.4	7.91	AM 1.5G / 1 Sun	0.97	2
GaN/Si	Cu	NiO	HCOOH, CH_4, C_2H_4, C_2H_5OH	35	1.58	AM 1.5G / 8 Suns	0.046	3
$SrTiO_3$	RuCP	W/O	$HCOO^-$	N.A.	0.15	λ < 400 nm / 1 Sun	0.14	4
TiO_2	Sn	W/O	$HCOO^-$	65	3.4	AM 1.5G / 2 Sun	0.24	5
$WO_3/BiVO_4$	$[NH_2C_3MIm][Br]$	W/O	$HCOO^-$	94.1	5.1	AM 1.5G / 1 Sun	0.11	6
TiO_2/CdS	Chitosan	W/O	CH_4	94.4	2	AM 1.5G / 1 Sun	1.28	7
$WO_3/BiVO_4$	Ag	W/O	CO	72	8.2	AM 1.5G / 1 Sun	0.53	8
$BiVO_4$	Meso ITO	FeOOH	$HCOO^-$	N.A.	0.6	λ > 400 nm / 1 Sun	0.03	9
**Si**	**Ag**	**Ni**	**CO**	**90**	**36**	**AM 1.5G / 1 Sun**	**2.42**	**This work**

Table S2 Performance comparison of photocathodes promoted CO_2RR

(Photo)cathode	Anode	Catalyst	Reduction products	FE (%)	Current Density (mA/cm^2)	Irradiation Conditions / Intensity	STF (%)	Reference
ZnTe/ZnO	Pt	Au	CO	35	1.5	AM 1.5G / 1 Sun	0.00525[a]	10
Si	Graphite	Au	CO	91	1.46	AM 1.5G / 1 Sun	0.106 [a]	11
GaN/Si	Pt	Cu-ZnO	CO	70	1	Xenon lamp / 8 Suns	0.126 [a]	12
GaN/Si	Pt	Pt	CO	78	4	Xenon lamp / 8 Suns	0.870	13
GaN/Si	Pt	Sn	HCOOH	76.9	1	AM 1.5G / 1 Sun	0.0369 [a]	14
a-Si	Pt	Au	CO	50	0.5	AM 1.5G / 1 Sun	0.42	15
GaN/Si	Pt	AuPt	CO	65	18	AM 1.5G / 1 Sun	1.88	16
$(CuGa)_{0.5}ZnS_2$	Pt	Bare	CO	21	0.1	λ > 400 nm / 3 Suns	0.006 [a]	17
Cu_2O	N.A.	Amine-functionalized polysiloxane	$HCOO^-$	61	2	N.A.	0.11	18
InP	Pt	Au-TiO_2	CO	84.2	4.5	AM 1.5G / 1 Sun	0.32	19
**Ag**	**Si**	**Ni**	**CO**	**90**	**36**	**AM 1.5G / 1 Sun**	**2.42**	**This work**

a: Estimated from J-V curve

Supplementary References

1. Deguchi M, Yotsuhashi S, Hashiba H, Yamada Y, Ohkawa K. Enhanced capability of photoelectrochemical CO_2 conversion system using an AlGaN/GaN photoelectrode. *Jpn. J. Appl. Phys.* **52**, 08JF07 (2013).
2. Sekimoto T, *et al.* Tandem photo-electrode of InGaN with two Si p-n junctions for CO_2 conversion to HCOOH with the efficiency greater than biological photosynthesis. *Appl. Phys. Lett.* **106**, 073902 (2015).
3. Yotsuhashi S, Deguchi M, Yamada Y, Ohkawa K. Effect of inserted Si p-n junction on GaN-based photo-electrochemical CO_2 conversion system. *AIP Adv.* **4**, 067135 (2014).
4. Arai T, Sato S, Kajino T, Morikawa T. Solar CO_2 reduction using H_2O by a semiconductor/metal-complex hybrid photocatalyst: enhanced efficiency and demonstration of a wireless system using $SrTiO_3$ photoanodes. *Energy Environ. Sci.* **6**, 1274 (2013).
5. Irtem E, *et al.* A photoelectrochemical flow cell design for the efficient CO_2 conversion to fuels. *Electrochim. Acta* **240**, 225-230 (2017).
6. Lu W, *et al.* Efficient photoelectrochemical reduction of carbon dioxide to formic acid: a functionalized ionic liquid as an absorbent and electrolyte. *Angew. Chem. Int. Ed.* **56**, 11851-11854 (2017).
7. Xiao S, *et al.* Hybrid microbial photoelectrochemical system reduces CO_2 to CH_4 with 1.28% solar energy conversion efficiency. *Chem. Eng. J.* **390**, 124530 (2020).
8. Lu W, Zhang Y, Zhang J, Xu P. Reduction of gas CO_2 to CO with high selectivity by Ag nanocube-based membrane cathodes in a photoelectrochemical system. *Ind. Eng. Chem. Res.* **59**, 5536-5545 (2020).
9. Kim J, *et al.* Robust FeOOH/$BiVO_4$/Cu(In, Ga)Se_2 tandem structure for solar-powered biocatalytic CO_2 reduction. *J. Mater. Chem. A* **8**, 8496-8502 (2020).
10. Jang YJ, *et al.* Selective CO production by Au coupled ZnTe/ZnO in the photoelectrochemical CO_2 reduction system. *Energy Environ. Sci.* **8**, 3597-3604 (2015).
11. Song JT, *et al.* Nanoporous Au thin films on Si photoelectrodes for selective and efficient photoelectrochemical CO_2 reduction. *Adv. Energy Mater.* **7**, 1601103 (2017).
12. Chu S, *et al.* Tunable syngas production from CO_2 and H_2O in an aqueous photoelectrochemical cell. *Angew. Chem. Int. Ed.* **55**, 14262-14266 (2016).
13. Chu S, *et al.* Photoelectrochemical CO_2 reduction into syngas with the metal/oxide interface. *J. Am. Chem. Soc.* **140**, 7869-7877 (2018).
14. Zhou B, *et al.* A GaN:Sn nanoarchitecture integrated on a silicon platform for converting CO_2 to HCOOH by photoelectrocatalysis. *Energy Environ. Sci.* **12**, 2842-2848 (2019).
15. Li C, *et al.* Photoelectrochemical CO_2 reduction to adjustable syngas on grain-boundary-mediated a-Si/TiO_2/Au photocathodes with low onset potentials. *Energy Environ. Sci.* **12**, 923-928 (2019).
16. Chu S, *et al.* Decoupling strategy for enhanced syngas generation from

photoelectrochemical CO_2 reduction. *iScience* **23**, 101390 (2020).

17. Yoshino S, Iwase A, Yamaguchi Y, Suzuki TM, Morikawa T, Kudo A. Photocatalytic CO_2 reduction using water as an electron donor under visible light irradiation by Z-scheme and photoelectrochemical systems over $(CuGa)_{0.5}ZnS_2$ in the presence of basic additives. *J. Am. Chem. Soc.* **144**, 2323-2332 (2022).
18. Li C, *et al.* Construction of heterostructured $Sn/TiO_2/Si$ photocathode for efficient photoelectrochemical CO_2 reduction. *ChemSusChem* **15**, e202200188 (2022).
19. Liu G, *et al.* Manipulating intermediates at the Au-TiO_2 interface over InP nanopillar array for photoelectrochemical CO_2 reduction. *ACS Catal.* **11**, 11416-11428 (2021).
